# Phenolics Profile and Phenol-Related Enzyme Activities in Cucumber Plants Under Ni Stress

**DOI:** 10.3390/ijms26031237

**Published:** 2025-01-31

**Authors:** Ewa Gajewska, Aleksandra Witusińska, Andrzej Kornaś, Marzena Wielanek

**Affiliations:** 1 Faculty of Biology and Environmental Protection, Department of Plant Physiology and Biochemistry, University of Lodz, Banacha 12/16, 90-237 Łódź, Poland; aleksandra.witusinska@biol.uni.lodz.pl (A.W.); marzena.wielanek@biol.uni.lodz.pl (M.W.); 2 Institute of Biology and Earth Sciences, University of the National Education Commission, Podchorążych 2, 30-084 Kraków, Poland; andrzej.kornas@uken.krakow.pl

**Keywords:** chlorophyll *a* fluorescence, flavonoids, lignin, nickel, phenolic acids, peroxidase

## Abstract

Ni phytotoxicity has been attributed to its multidirectional detrimental effects on plant cell structure and function. However, relatively little is known about Ni’s impact on phenolic metabolism in plants. The objective of our study was to obtain insight into the effect of Ni treatment on phenolic compound composition, phenol-related enzyme activities, and lignin accumulation in cucumber plants. Besides growth reduction, the chlorophyll *a* and carotenoid contents as well as the chlorophyll *a* fluorescence parameters, namely, the maximum photochemical efficiency of PS II and non-photochemical quenching, were significantly decreased in the Ni-treated cucumber plants. Application of Ni resulted in changes in the phenolic acid and flavonoid profiles; however, the total content of the detected phenolic compounds remained unchanged in the leaf and slightly decreased in the root. The Ni-induced release of free phenolic acids from their conjugates was found in the leaf. Ni treatment led to a marked increase in leaf peroxidase activities assayed with various phenolic substrates, while it did not influence phenyl ammonia lyase and polyphenol oxidase activities. Increased lignin deposition was observed in the leaf blade of Ni-exposed plants. Neither lignin accumulation nor induction of peroxidase activities were found in the root. Our results indicate that the Ni effect on phenolic compound composition and related enzyme activities is organ-specific. The observed changes in the content of individual compounds might result rather from the metal-triggered conversions of the compounds constitutively present in the cucumber tissues than from de novo synthesis.

## 1. Introduction

Nickel has been classified as a microelement; however, its excessive concentrations become toxic to most plant species. The toxicity of this heavy metal is evidenced by the appearance of various symptoms including growth reduction, wilting, leaf chlorosis, or root browning. Nickel’s phytotoxicity has been attributed to its detrimental impact on metabolic pathways and key physiological processes [1]. Compared to other heavy metals, Ni’s effect on plants has been studied less intensively. There are only a few reports on the effect of Ni on phenol metabolism [2,3].

Phenolic compounds are secondary metabolites widely distributed in plants. Their structure is characterized by the presence of at least one aromatic ring bearing one or more hydroxyl groups [4]. Phenolics originate from L-phenylalanine produced in the shikimate pathway. Conversion of L-phenylalanine to *trans*-cinnamic acid catalyzed by L-phenylalanine ammonia lyase (PAL, EC 3.5.1.5) is the first step in the biosynthesis of phenolic compounds. This heterogenous group encompasses, among others, simple phenols, phenolic acids, and flavonoids [5]. Both free and conjugated forms of phenolic compounds can be found in plants. The most common phenolic conjugates are β-O-glycosides in which one or more sugar moieties are linked to the OH group [6]. Due to the presence of hydroxyl groups in their structure, phenolics show antioxidant properties. These compounds are believed to prevent oxidative stress as direct reactive oxygen species (ROS) scavengers and electron donors for antioxidative enzymes. It is considered that the antioxidative activity of phenolic compounds depends on the number and position of OH groups [4,7]. The protective properties of phenolic compounds under heavy metal stress can be also ascribed to their metal chelation ability [8].

Peroxidases (POXs, EC 1.11.1.7) use $H_{2}O_{2}$ to oxidize a variety of organic compounds including phenols. These enzymes are considered to participate in processes of cell wall stiffening, namely, lignification and cross-linking of cell wall polymers. Being involved in the remodeling of cell walls, POXs play an important role in plants under both physiological and stress conditions [9,10]. POX activity is usually measured using guaiacol as a universal substrate. It has been proposed that the application of more specific substrates such as syringaldazine, phenolic acids, or flavonoids in a POX activity assay may help us to understand better the role of POXs in individual processes [11]. It is believed that POXs participating in lignin synthesis in vitro display high affinity toward syringaldazine, a lignin monomer analog [12]. Determination of POX activity with ferulic acid is assumed to reflect the dynamics of POX-mediated oxidation of this phenolic acid, resulting in the formation of diferulate bridges between cell wall polysaccharides [13,14]. Peroxidase using quercetin as a substrate is considered to play a significant role in the removal of $H_{2}O_{2}$, thus supporting other antioxidant enzymes [15].

Polyphenol oxidases (PPOs) catalyze the oxidation of phenols to quinones using molecular oxygen as an electron acceptor. According to the substrate specificity and mechanism of reaction, PPOs are divided into several groups including laccases (PPOL, EC 10.3.2) that oxidize both o-diphenols and *p*-diphenols to the corresponding quinones and catechol oxidases (PPOC, EC 1.10.3.1), which are able to oxidize solely *o*-diphenols. It is believed that PPOs cooperate with POXs in lignin synthesis and cross-linking of cell wall components [16].

Lignin is a complex component of the plant cell wall consisting of phenolic heteropolymers which are covalently bound to both polysaccharides and proteins. Lignin is synthesized from three *p*-hydroxycinnamyl alcohols, namely, *p*-coumaryl, coniferyl, and sinapyl alcohol, collectively called monolignols. In the first step, they are enzymatically oxidized to the corresponding oxyradicals, which subsequently spontaneously polymerize, forming lignin [17].

The cucumber is a widely cultivated and economically important crop, which in our earlier research was shown to be relatively sensitive to Ni [18]. The appearance of necrotic spots on the oldest (the first) leaf of Ni-treated cucumber plants suggested increased lignification and possible alterations in the metabolism of phenolics. Therefore, the objective of our study was to obtain insight into the Ni effect on phenolic compound composition in cucumber plants including a separate analysis of free and conjugated compounds. The activities of enzymes involved in phenolic synthesis or oxidation as well as parameters indicating the stress level in plants were studied in parallel. To our knowledge, this is the first report presenting the Ni effect on the ratio of free to conjugated forms of phenolic compounds and showing the Ni-induced changes in the flavonoid profile.

## 2. Results

### 2.1. Growth, Ni Accumulation, and Relative Water Content (RWC)

**Figure 1:**
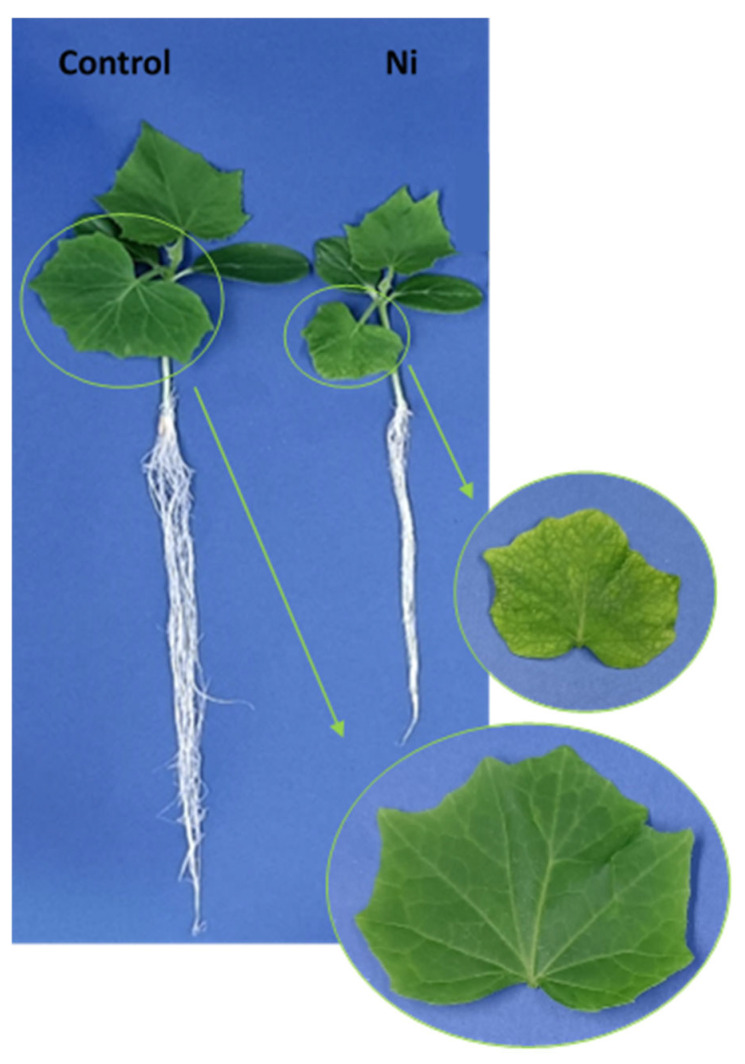
Appearance of the 14-day-old control and Ni-treated cucumber plants.

Nickel treatment caused significant growth inhibition of both the aboveground and underground parts of cucumber plants (Figure 1). A considerable reduction in root length and a decrease in the number of lateral roots were observed compared to the control. In addition to the reduced leaf blade surface area, chlorosis and small necrotic spots were visible on the first (the oldest) leaf, which was taken for further analyses.

Nickel treatment markedly reduced the fresh weight of the leaf and root by 53% and 59%, respectively (Table 1). Accumulation of the metal in the root was about 2-fold higher compared to the leaf. No significant changes in RWC were observed in the Ni-treated cucumber plants.

### 2.2. Photosynthetic Pigments and Chlorophyll a Fluorescence

**Figure 2:**
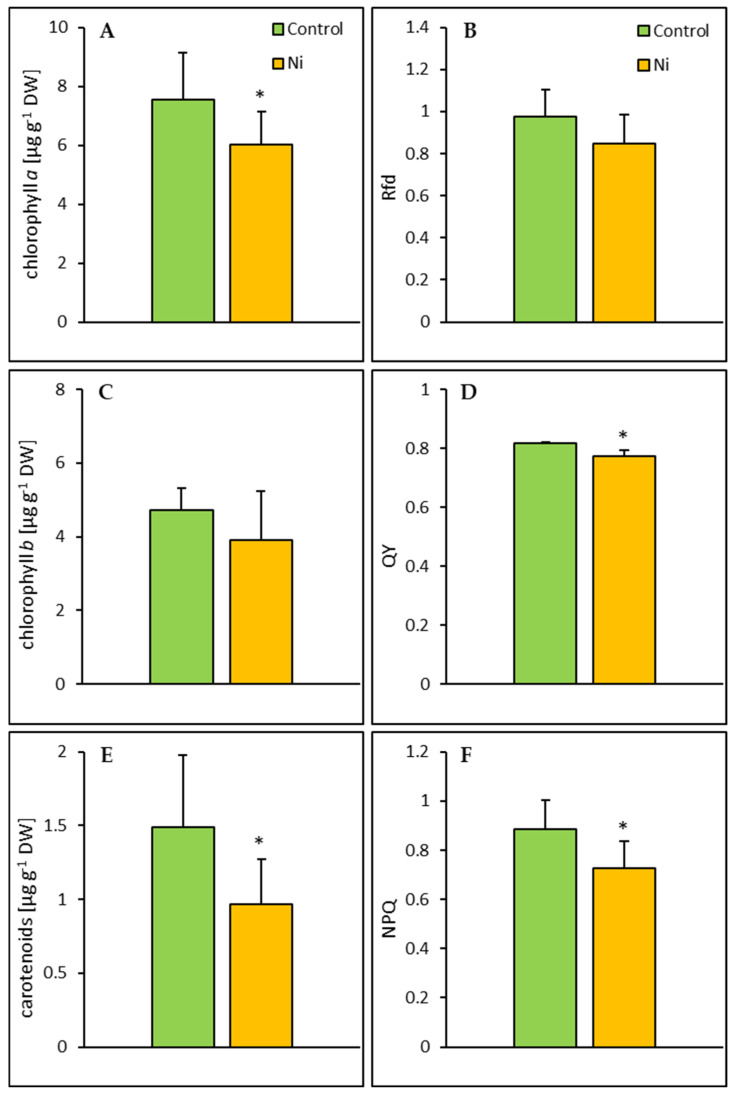
Effect of Ni treatment on chlorophyll *a* (**A**), chlorophyll *b* (**C**), and carotenoid (**E**) contents, fluorescence decline ratio (Rfd) (**B**), maximum photochemical efficiency of PS II (QY) (**D**), and non-photochemical quenching (NPQ) (**F**) in the cucumber leaf. Data represent mean values ± SD (n = 6–9); * indicates values that differ significantly from the control at *p* ˂ 0.05.

Exposure of cucumber plants to Ni resulted in a significant decrease in chlorophyll *a* and carotenoid contents by 20% and 35%, respectively (Figure 2). The maximum photochemical efficiency of PS II and non-photochemical quenching values in the leaf of Ni-treated cucumber plants were 6% and 18% lower than in the control, respectively.

### 2.3. Phenolic Compound Composition Analysis

**Figure 3:**
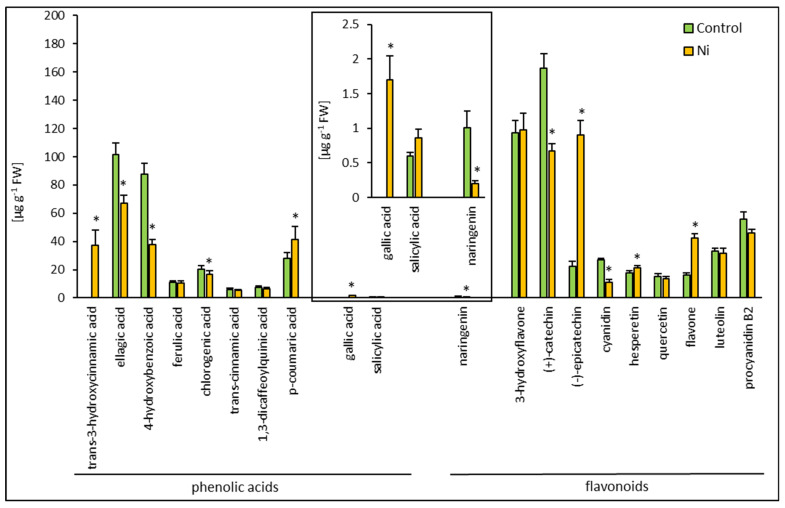
Effect of Ni treatment on phenolic compounds composition in cucumber leaf. Data represent mean values ± SD (n = 3); * indicates values that differ significantly from the control at *p* ˂ 0.05.

**Figure 4:**
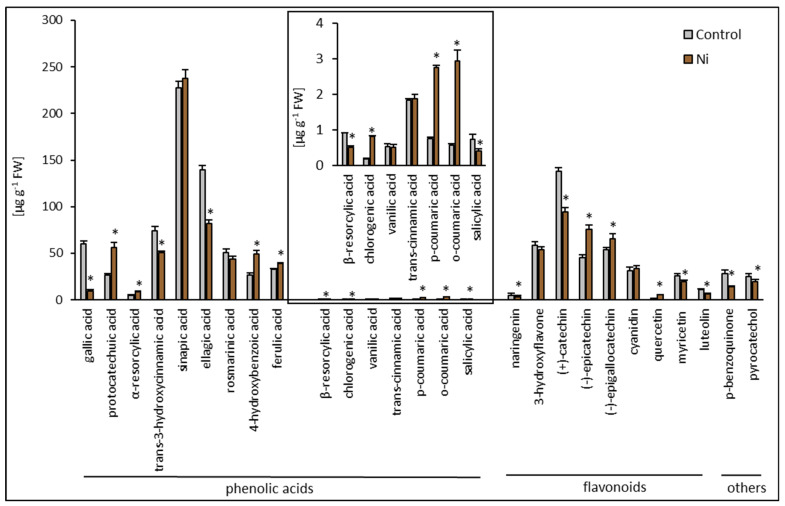
Effect of Ni treatment on phenolic compound composition in cucumber root. Data represent mean values ± SD (n = 3); * indicates values that differ significantly from the control at *p* ˂ 0.05.

HPLC analysis revealed a distinct constitutive phenolic compound composition of the cucumber leaf and root. Generally, in the leaf, 20 compounds were detected (Figure 3), while in the root, 27 phenolic compounds were found (Figure 4).

Among the phenolic compounds in the leaf, 10 phenolic acids and 10 flavonoids were detected. Cinnamic acid derivatives included *trans*-3-hydroxycinnamic acid, ferulic acid, chlorogenic acid, *trans*-cinnamic acid, and 1,3-dicaffeoylquinic acid. Benzoic acid derivatives encompassed ellagic acid, 4-hydroxybenzoic acid, *p*-coumaric acid, gallic acid, and salicylic acid. Flavonoids detected in the cucumber leaf included 3-hydroxyflavone, flavone, (+)-catechin, (−)-epicatechin, cyanidin, naringenin, hesperetin, quercetin, luteolin, and procyanidin B2. The most abundant phenolic acids were ellagic acid (39%) and 4-hydroxybenzoic acid (33%). The most abundant flavonoids were (+)-catechin (35%) and 3-hydroxyflavone (25%) (Figure 3). Among the phenolic compounds in the root, 16 phenolic acids and 11 flavonoids were detected. Cinnamic acid derivatives included *trans*-3-hydroxycinnamic acid, sinapic acid, rosmarinic acid, ferulic acid, chlorogenic acid, and trans-cinnamic acid. Benzoic acid derivatives encompassed gallic acid, protocatechuic acid, *α*-resorcylic acid, *β*-resorcylic acid, ellagic acid, 4-hydroxybenzoic acid, vanillic acid, *p*-coumaric acid, *o*-coumaric acid, and salicylic acid. Flavonoids detected in the cucumber leaf included myricetin, 3-hydroxyflavone, (+)-catechin, (−)-epicatechin, (−)-epigallocatechin, cyanidin, naringenin, quercetin, and luteolin. Apart from the abovementioned phenolic acids and flavonoids, *p*-benzoquinone and pyrocatechol were found in the root extract. The most abundant phenolic acids in the root were sinapic acid (30%) and ellagic acid (22%), while the most abundant flavonoid was (+)-catechin (37%) (Figure 4). In general, the leaf and root of the control cucumber plants differed in the total phenolic acid and flavonoid contents. A 2.5-fold lower content of phenolic acids and a 1.3-fold higher content of flavonoids were found in the leaf compared to the root.

Considering all phenolic compounds in the profile, Ni did not cause any change in the total phenolic compound content in the cucumber leaf. Exposure to Ni resulted in the appearance of two new compounds, absent in the control, i.e., gallic acid and *trans*-3-hydroxycinnamic acid in the leaf. The most pronounced increases were found for (−)-epicatechin and flavone contents, with about 5-fold and 2.6-fold enhancement, respectively. Increased contents of *p*-coumaric acid and hesperetin were also observed in the leaf of cucumber plants treated with Ni. Decreases in the content of 4-hydroxybenzoic acid, ellagic acid, and chlorogenic acid, as well as naringenin, cyanidin, and (+)-catechin, were also noted (Figure 3). Nickel treatment caused a slight reduction (about 6%) in the content of phenolic compounds in the root of cucumber plants. The highest values, a 5.2-fold increase in *o*-coumaric acid content and a 4.3-fold increase in chlorogenic acid, were observed in the root. Increases in the contents of *p*-coumaric acid, quercetin, and protocatechuic acid were also found. Smaller increases in the content were noted for 4-hydroxybenzoic acid, α-resorcylic acid, (−)-epicatechin, (−)-epigallocatechin, and ferulic acid. Decreases in the contents of gallic acid, *p*-benzoquinone, *β*-resorcylic acid, salicylic acid, ellagic acid, luteolin, naringenin, (+)-catechin, *trans*-3-hydroxycinnamic acid, myricetin, and pyrocatechol were found (Figure 4).

In order to analyze the content of phenolic compounds more precisely, acid hydrolysis of the extracts was performed, which allowed the separation of pools of free and conjugated phenolic compounds. The results concerning the Ni effect on free and conjugated phenol contents in the cucumber leaf and root are shown in Table 2 and Table 3, respectively.

In the control leaf, (−)-epicatechin occurred mainly in the free form (96.5%), while hesperetin was present mainly in the conjugated form (86.4%). Quercetin and luteolin occurred only in the conjugated form (Table 2). In the root, gallic acid was present mainly in the free form (99.1%), while ferulic acid occurred mainly in the conjugated form (96.5%). Similarly, quercetin occurred only in the conjugated form in the root of cucumber plants (Table 3).

Taking into account the entire pool of phenolic acids in the leaf, the content of their free forms increased about 1.6-fold as a result of Ni treatment. The largest increases in free forms, about 2-fold, were found for 4-hydroxybenzoic acid and ellagic acid. However in the case of most phenolic acids, Ni did not significantly influence the ratio of free to conjugated forms. As for the entire leaf flavonoid pool, the ratio of their free forms to their total content did not change after Ni treatment. However, the content of free procyanidin B2 increased by over 2.5-fold, while the content of free (−)-epicatechin and hesperetin decreased by 67% and 34%, respectively. In the root, increased release from conjugated forms was found for 10 phenolic compounds, with the largest, more than 8-fold, for ferulic acid, as well as *trans*-cinnamic acid (2.7-fold) and pyrocatechol (2.7-fold). A significant decrease in the abundance of free forms of phenolic compounds in the total pool was observed for seven phenolic compounds, the largest of which was observed for (−)-epigallocatechin (75%), (−)-epicatechin (51%), and *o*-coumaric acid (51%).

### 2.4. Enzyme Activities

**Figure 5:**
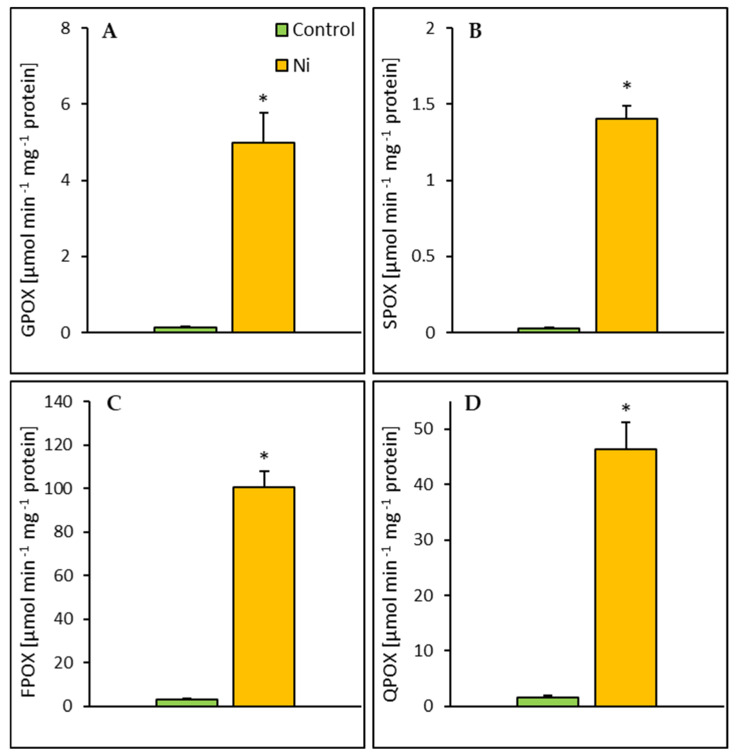
Effect of Ni treatment on the activity of POX assayed with guaiacol (GPOX) (**A**), syringaldazine (SPOX) (**B**), ferulic acid (FPOX) (**C**), and quercetin (QPOX) (**D**) in the cucumber leaf. Data represent mean values ± SD (n = 3–7); * indicates values that differ significantly from the control at *p* ˂ 0.05.

**Figure 6:**
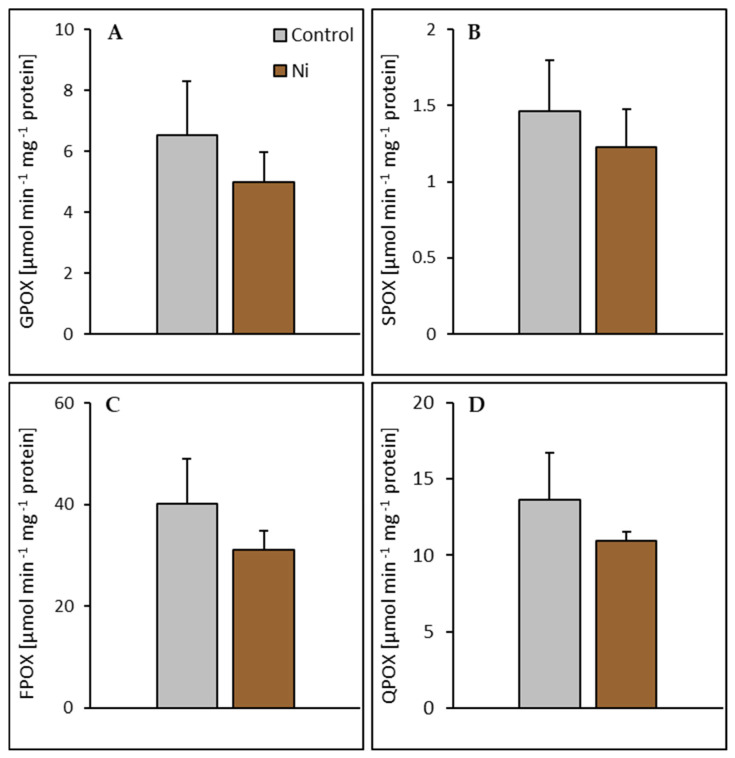
Effect of Ni treatment on the activity of POX assayed with guaiacol (GPOX) (**A**), syringaldazine (SPOX) (**B**), ferulic acid (FPOX) (**C**), and quercetin (QPOX) (**D**) in the cucumber root. Data represent mean values ± SD (n = 3–7).

In the leaf, Ni treatment caused a significant increase in POX activities assayed with all tested phenolic substrates: guaiacol (GPOX), syringaldazine (SPOX), ferulic acid (FPOX), and quercetin (QPOX) (Figure 5). Exposure of cucumber plants to Ni led to an increase in POX activities in the range of 30–47-fold depending on the substrate used. The highest activity increase was found for SPOX and the lowest was for QPOX. No significant changes in POX activities were noted in the root (Figure 6).

**Figure 7:**
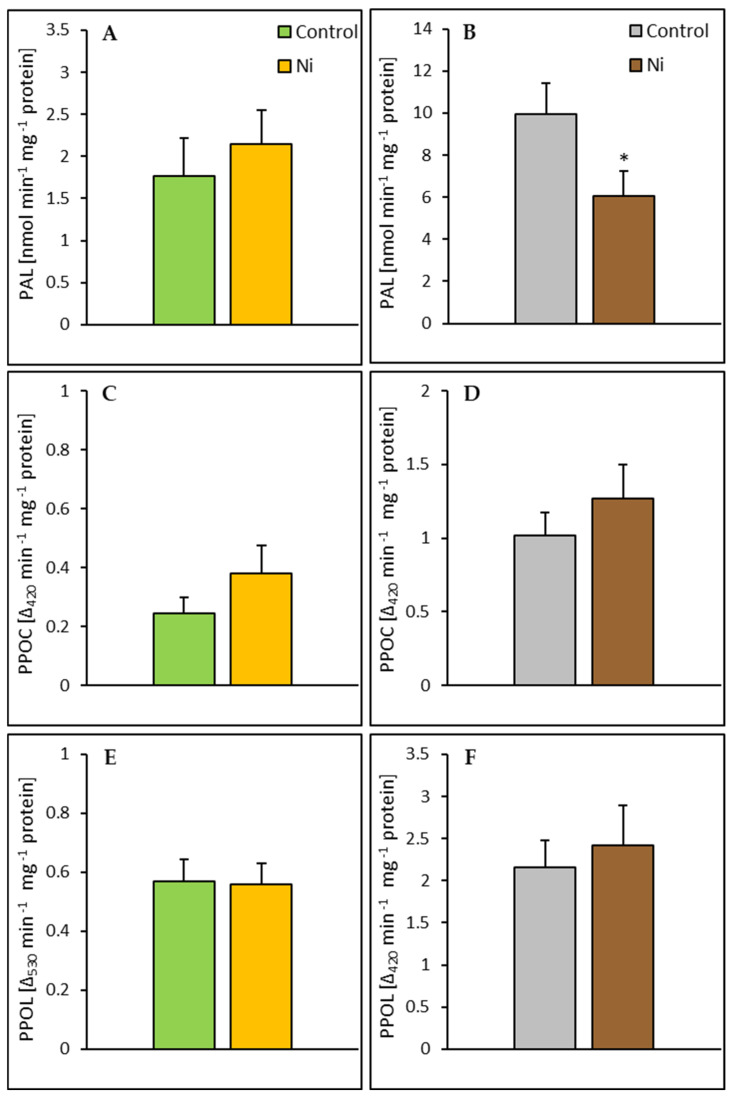
Effect of Ni treatment on PAL, PPOC, and PPOL activities in cucumber leaf (**A**,**C**,**E**) and root (**B**,**D**,**F**). Data represent mean values ± SD (n = 3–7); * indicates values that differ significantly from the control at *p* ˂ 0.05.

Exposure of cucumber plants to Ni resulted in a 39% decrease in PAL activity in the root but did not significantly affect the activity of this enzyme in the leaf. In both the leaf and the root, Ni application did not significantly alter the activities of PPOC and PPOL (Figure 7).

### 2.5. Native Polyacrylamide Gel Electrophoresis (PAGE)

**Figure 8:**
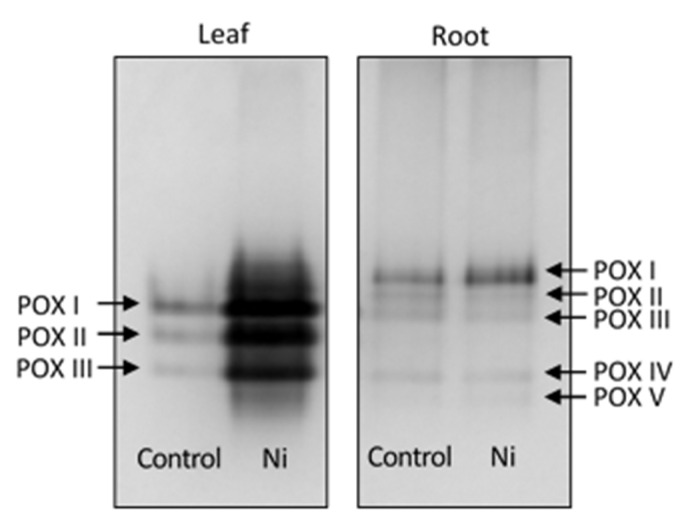
Leaf and root peroxidase isoforms from the control and the Ni-treated cucumber plant.

Using electrophoretic protein separation, the differentiation of POX isoenzymatic forms was demonstrated. In the leaf of the control cucumber plants, three POX isoforms were detected, while in the root, five POX isoforms were found (Figure 8). Ni treatment led to an increase in the intensity of the bands constitutively present in the control. No new POX isoforms appeared after Ni treatment.

### 2.6. Histochemical Detection of Lignin

**Figure 9:**
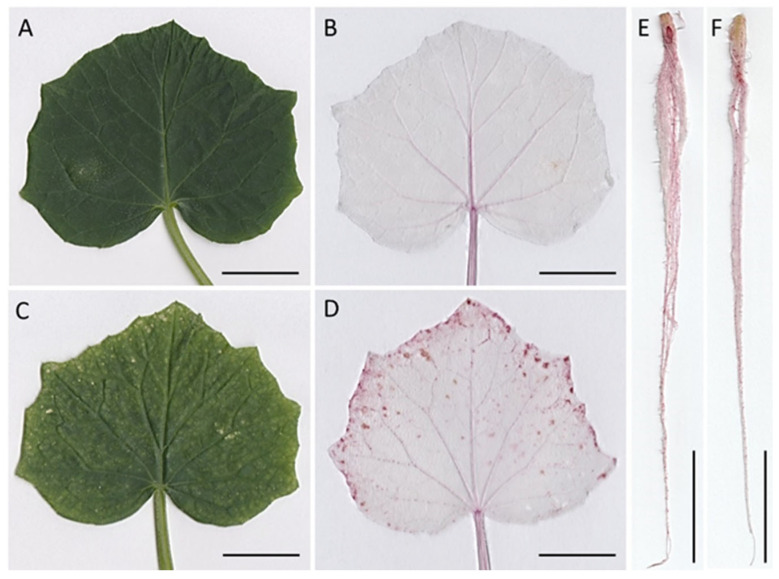
Effect of Ni treatment on lignin content in cucumber leaf (**A**–**D**) and root (**E**,**F**). **A**, **C**—control and Ni-treated leaf before chlorophyll removal and phloroglucinol staining; (**B**,**D**)—control and Ni-treated leaf after phloroglucinol staining; (**E**,**F**)—control and Ni-treated root after phloroglucinol staining. Bar = 2 cm.

In situ detection of lignin in cucumber leaf and root was performed using phloroglucinol staining (Figure 9). Nickel treatment caused a significant lignin accumulation in the leaf. While in the control leaf lignin was mainly located in the veins, in the leaf of cucumber seedlings treated with Ni, pink spots indicating the presence of lignin were observed in interveinal areas, mainly at the edge of the blade. The roots of the control and Ni-treated plants showed no visible differences in lignin accumulation.

## 3. Discussion

Growth inhibition is a common plant response to stress, including that caused by heavy metals. In accordance with the results of previous studies on the Ni effect on plants [19,20,21], we found over a 50% decrease in the fresh weight of cucumber seedlings, with the root being slightly more affected compared to the leaf. The stronger inhibition of root growth could be related to the more-than-twice-higher Ni accumulation in this organ compared to the leaf. Higher Ni accumulation in roots was also found in wheat [22] and peanuts [23]. Accumulation of most of the uptaken metal in the underground part is a typical reaction of excluder plant species, including cucumber [24]. This is considered a plant defense strategy aimed at protecting the photosynthetic apparatus against the toxic effects of metal [25].

Reduction in plant growth under Ni stress may result from disruption of metabolic pathways and physiological processes by the metal. Our previous studies conducted on wheat seedlings revealed the Ni-triggered disturbance in nitrogen and carbon metabolism [26,27]. Although growth suppression is usually considered to be a consequence of stress, it has also been proposed to be, at least partly, a strategy of adaptation to stress conditions [28]. The existence of such a mechanism in cucumber plants subjected to Ni stress cannot be excluded.

Nickel has been reported to affect water relations in plants, which was evidenced by decreased RWC [29,30]. In contrast, in our study, Ni treatment did not significantly influence RWC in either the leaf or the root. The possible explanation for this result could be that the applied Ni dose was not high enough to affect water relations in the leaf or root tissues.

Apart from growth parameters, photosynthetic pigment contents and chlorophyll *a* fluorescence are often measured to assess stress level in plants. The total chlorophyll content in the cucumber leaf decreased in response to Ni treatment; however, only in the case of chlorophyll *a* did this reduction occur at a statistically significant level. Reduction of chlorophyll content in response to stress factors, including metals, may be the result of its reduced biosynthesis [31] or increased degradation [32]. It is possible that the reduction in chlorophyll content observed in our study was also associated with a significant decrease in carotenoid concentration. It is believed that carotenoids, as non-enzymatic antioxidants, play an important role in protecting chlorophyll against oxidative stress [33]. A decrease in carotenoid concentration in response to Ni treatment was also found in pumpkin leaves [34].

The fluorescence-decline ratio is considered to be an excellent vitality index and indicator of stress condition in plants [35]. In our study, the Rfd value remained unchanged, which could suggest that Ni treatment did not affect the vitality of cucumber plants. Contrary to our findings, exposure of green filamentous alga *Zygnema* sp. to Cd and Zn led to a decrease in Rfd [36]. Although Rfd was not altered in the Ni-treated cucumber, the QY, reflecting the maximum photochemical efficiency of photosystem II (PSII), was significantly decreased. A decline in QY was previously reported for Ni-treated wheat seedlings [37]. Disruption of the PSII functioning under Ni-stress conditions may result from photoinhibition in the PSII reaction center induced by this metal, as demonstrated by Jahan et al. [38]. Reduction in QY was also observed in plants exposed to Cu [39], Cd, and Pb [40]. Non-photochemical quenching (NPQ) based on thermal dissipation of excess light energy is thought to be an important process preventing photoinhibition in plants subjected to stress [41]. Decreased NPQ found in our study indicates that the NPQ-dependent energy dissipation mechanism was affected by Ni stress. This could be related to a pronounced decrease in carotenoid content in the cucumber leaf. Xanthophylls, which are a type of carotenoids, are involved in the protection of the photosynthetic apparatus by dissipating excessive excitation energy in the form of heat. Consistent with our results, NPQ decline was also observed in mulberry leaves after Pb and Cd application [40]. However, increases in NPQ in response to heavy metal treatment were also reported [39,41]. Our results suggest that QY and NPQ are better than Rfd indicators of a stress condition in Ni-treated cucumber plants.

It has been reported that overexcitation of the photosynthetic apparatus may result in ROS formation [41]. Our previous study conducted on Ni-stressed cucumber plants revealed a marked accumulation of $O_{2}^{.-}$ and $H_{2}O_{2}$ in the leaf [18]. It can be suggested that the observed enhancement of ROS levels in cucumber leaves could have been, at least partly, related to the Ni-induced disturbance of the NPQ mechanism.

Phenolic compounds are considered to play an important protective role in plants under stress conditions [5]. It has been suggested that the concentration of phenolics in plant tissues may be used to assess the impact of environmental stress in plants [42]. HPLC analysis revealed distinct constitutive phenolic profiles in the cucumber leaf and root. Compared to the leaf, in the root more compounds, mainly phenolic acids, were detected. In the root, 16 phenolic acids were found, while in the leaf, only 8 phenolic acids were present. With regard to flavonoids, a similar number of compounds were detected in both organs. Some compounds occurred exclusively in the leaf or in the root. Apart from the qualitative differences in phenolic composition, the organs also differed in the quantity of individual compounds. Among phenolic acids, in the leaf, ellagic acid was the most abundant, while in the roots, sinapic acid was the dominating compound. However, in both organs, (+)-catechin was the most abundant flavonoid. Variation in the phenolic compound composition between leaves and roots was also reported for other plant species. According to the literature data, phenolic compound composition is both species- and organ-specific [3,43,44].

Nickel treatment generally did not influence the total contents of phenolic acids and flavonoids detected in the leaf and slightly decreased them in the root. However, changes were found in the content of most individual compounds. Both increases and decreases were observed in phenolic contents after Ni treatment. In the leaf, the most pronounced increase was shown for (−)-epicatechin and the strongest decrease for naringenin. Two new compounds, absent in the control, namely, *trans*-3-hydroxycinnamic acid and gallic acid, appeared in the leaf in response to Ni treatment. The emergence of new compounds was also reported for the leaves of *Basilicum polystachyon* treated with Pb and Hg [45]. The appearance of gallic acid in the leaf of cucumber plants exposed to Ni was accompanied by a decrease in ellagic acid content, which may imply the metal-induced degradation of ellagic acid, a dimeric form of gallic acid. It is also possible that the increase in (−)-epicatechin content could have resulted from the epimerization of (−)-catechin, whose content was significantly decreased in response to Ni treatment. In the root chlorogenic, *p*-coumaric and *o*-coumaric acids, being the less abundant in the control plant, showed the most pronounced increases in response to Ni application. The most affected was the gallic acid content. Similarly to the leaf, Ni-evoked conversion of (−)-catechin to (−)- epicatechin can be suggested. In accordance with our findings, the total phenolic acid content remained unchanged in the leaves of Ni-treated *Matricaria chamomilla* plants. However, in contrast to the cucumber plants, the *M. chamomilla* roots responded to Ni stress with an accumulation of phenolic acids [3].

Accumulation of phenolic compounds in the tissues of Ni-treated cucumber plants may be related to their involvement in defense against Ni stress. Phenols are known for their antioxidant properties. The most important determinants for the antioxidant potential of phenols are the number and localization of OH groups. Flavonoids’ antioxidant activity additionally depends on the presence of a 3 OH group in the C ring and a 5 OH group in the A ring as well as the presence of the orto-3’,4’-dihydroxy structure in the B ring [4]. Apart from antioxidant activity, phenolic compounds are also good metal chelators [8]. Ni^2+^ ions were shown to bind preferentially to carboxyl groups of hydroxycinnamic acids [46].

Phenolic compounds, similar to other secondary metabolites, often occur in plants as glycoconjugates. Most phenolics are nucleophilic molecules with high damage potential to cellular components. It is believed that storing phenolic compounds in the form of inactive conjugates is a common strategy to protect plants from their toxicity. Glycosylated phenolics are considered as a reserve of active compounds which can be mobilized under stress conditions. Moreover, glycosylation increases the stability and solubility of phenolics and thus is essential for their transport within plants [47,48]. To obtain better insight into the Ni effect on phenolic composition in cucumber plants, we analyzed leaf and root extracts before and after acid analysis, which is believed to degrade glycosidic bonds [45]. This allowed us to distinguish between free and glycosylated phenolic forms. Taking into account the total phenolic acid and flavonoid pools in the control leaf, the conjugated forms dominated. Similarly, in the control *B. polystachyon* leaves, mainly glycosides of phenolic acids were found [45]. In the cucumber root, free and conjugated forms of phenolic acids and flavonoids were present in similar quantities. Individual compounds showed various aglycon/glycoside ratios. Some of them occurred mainly as free forms and others mainly as glycosides. Quercetin in both organs and luteolin in the leaf were found exclusively as glycosylated forms.

Nickel treatment markedly influenced the ratio of the free to the conjugated form of individual compounds, resulting in both increases and decreases in the free-form content depending on the compound. In the leaf, generally, the amount of free phenolic acids increased in response to Ni treatment, indicating the Ni-induced degradation of conjugates. In line with our findings, stimulation of phenolic acid deglycosylation was reported for the leaves of *B. polystachyon* exposed to Pb and Hg. In the cucumber root, the deglycosylation degree of the total phenolic acids pool was lower compared to the leaf; however, a considerable release of the free forms of some compounds was found. The free ferulic acid content in the roots of Ni-treated cucumber plants was over 8-fold higher compared to the control. Taking into account the total flavonoid pool, the content of their aglycones in the leaf remained unaltered. Contrary to our results, an intensive release of isoflavonoid aglycones was reported for Cu-treated alfalfa seedlings [49]. A decrease in the root total free flavonoid content found in our study could have resulted mainly from the Ni-stimulated glycosylation of some individual compounds, including (−)-epicatechin and (−)-epigallocatechin, accounting for 40% of the total flavonoid content. The glycosylation status of phenolic compounds is regulated by the action of glycosyltransferases catalyzing the formation of glycoconjugates and glycoside hydrolases degrading the glycosidic bond between the phenol and sugar moiety [47]. Thus, the changes in the amount of free and conjugated phenolic forms observed in cucumber plants may suggest activation of these enzymes under the condition of Ni stress.

The release of phenolic compounds from their conjugated forms may improve their antioxidant and chelating activity. The glycosylated forms of phenols were found to exhibit lower antioxidant potential compared to their aglycones [50]. It is also believed that free phenolic compounds have higher complexing potential than the respective glycosides [6,8]. Deglycosylated forms of phenolics, especially flavonoids, may also serve as electron donors for POXs and thus can be involved in the removal of $H_{2}O_{2}$ [51].

Salicylic acid (SA) is an important signaling molecule, activating plant defense responses against both biotic and abiotic stresses [52,53]. In the pool of phenolic acids in cucumber plants, a small quantity of SA was found. In the leaf and the root, it accounted for 0.2% and 0.1% of the total phenolic acids content, respectively. A similar abundance of SA was also reported for *M. chamomilla* leaf and root tissue [3]. In cucumber plants, the total SA content was not significantly influenced by Ni treatment in the leaf, but in the root, it was significantly decreased. To our knowledge, only one paper dealing with the Ni effect on SA content has been published before [3]. Both increases and decreases in SA content were observed in the root and leaf of *M. chamomilla* plants depending on the Ni dose. In the present study, in both cucumber organs Ni treatment did not influence the ratio of free to conjugated SA forms. Both our findings and the results of the above-cited paper do not provide a clear answer regarding the role of endogenous SA in the plant response to Ni stress. However, an enhancement of Ni stress tolerance after the application of exogenous SA was demonstrated for mustard plants [54].

Phenolic biosynthesis begins with PAL-catalyzed deamination of L-phenylalanine. In our study, no significant modifications in PAL activity were found in the leaf, and in the root, it was decreased in response to Ni treatment. Unchanged PAL activity in the leaf corresponded with the unaltered content of *trans*-cinnamic acid, the direct product of the PAL-catalyzed reaction. In the roots, despite decreased PAL activity, *trans*-cinnamic acid content also did not show changes compared to the control. In the cucumber leaf, unaltered PAL activity was accompanied by the total content of detected phenolic compounds being unchanged. In the root, both PAL activity and phenolic compound content showed a decreasing trend in response to Ni application. Our results suggest that Ni stress did not induce de novo synthesis of phenolic compounds in cucumber plants. The observed changes in the content of individual compounds might have resulted from the metal-triggered conversions of the compounds constitutively present in the cucumber tissues. In agreement with the results of our study, unaltered or reduced PAL activity was found in the roots of Pb-treated soybean seedlings [55] and in the cotyledons of Ni-treated *Jatropha curcas* [2], respectively. However, increases in this enzyme activity in the responses of plants to heavy metal treatment have also been reported [55,56,57].

Enzymatic oxidation of phenolic compounds may be catalyzed by PPOs or POXs using $O_{2}$ or $H_{2}O_{2}$ as electron acceptors, respectively. In our study, both in the leaf and the root, neither PPOC nor PPOL activity was significantly influenced by Ni treatment. Similarly, unaltered PPO activity was found in the leaves of Ni-treated *M. chamomilla* [3] and Cd-treated *Brassica juncea* plants [58]. In contrast, a pronounced increase in PPO activity was observed in the leaves and roots of Cu- and Zn-treated *Kandelia obovata* plants [42]. A significant reduction in this enzyme activity was reported for the roots of Ni-treated *M. chamomilla* [3]. In our study, POX activities in the leaf increased markedly after Ni, while in the root no significant alterations in POX activities were observed. Similarly, Ni-induced several-fold enhancement in POX activity in the shoot accompanied by unchanged activity of this enzyme in the root was reported for wheat seedlings [59]. Depending on the substrate used in the assay, POX activity in the leaf of Ni-treated cucumber plants was 30–47-fold higher compared to the respective control. Apart from the widely used unspecific substrate guaiacol, we also measured POX activity using more specific substrates, namely, ferulic acid, and syringaldazine, which are considered to reflect the activities of POXs involved in plant cell wall strengthening processes, namely, diferulate bridge formation and lignin synthesis, respectively [12,14]. Increased FPOX and SPOX activities found in the cucumber leaf may suggest that Ni treatment activated the POX-mediated processes of cell wall reinforcement. A Ni-induced increase in SPOX activity accompanied by accumulation of lignin was demonstrated in the roots of rice seedlings [60].

Peroxidases using quercetin or other flavonoids as electron donors are believed to play an important role in the removal of $H_{2}O_{2}$ [15,51]. In the leaf of Ni-treated cucumber plants, we found a considerable increase in QPOX activity, which coincided with a significant accumulation of $H_{2}O_{2}$ reported in our previous work [18]. Since an enhancement in $H_{2}O_{2}$ level was accompanied by a decrease in CAT and only a slight increase in APX activities [18], it can be suggested that in the Ni-stressed cucumber leaf, QPOX may be involved in $H_{2}O_{2}$ detoxication, thereby supporting the main antioxidant enzymes degrading this ROS.

Plant peroxidases are known for the multiplicity of their isoforms [9]. To obtain insight into the effect of Ni on POX isoforms, non-denaturing PAGE of the leaf and the root proteins was performed. In the control plants, three POX isoforms were detected in the leaf and five isoforms in the root. In both organs, Ni treatment increased the intensity of POX bands already present in the control; however, the POX isoform pattern remained unchanged. Consistent with our results, an increase in individual isoform activities and lack of appearance of new isoforms were reported for cotyledons of Ni-treated *Jatropha curcas* [2]. In contrast, in garden cress sprouts, two new POX isoforms were found in response to Cd and Pb treatment [61].

Lignin is principally deposited in the secondary wall of certain plant tissues, including the xylem and phloem. Synthesis of this polymer may be also induced in response to biotic and abiotic stress. In plants exposed to heavy metals, lignin is considered to serve as the physical barrier against metal ions’ ingress into the cell [62]. Staining with phloroglucinol revealed a considerable accumulation of lignin in the leaf of the Ni-treated cucumber plant. Lignin distribution in the leaf blade generally corresponded with the pattern of necrotic spots that appeared in response to Ni treatment. Accumulation of lignin accompanied by an increase in SPOX activity implies the Ni-promoted POX-dependent lignification in the cucumber leaf. There are some previous reports on an enhancement in lignin content in response to heavy metals [39,56,63]; however, to our knowledge, the phloroglucinol-based in situ lignin detection in heavy metal-stressed plants has not been presented before. Contrary to the leaf, in the cucumber root neither enhanced lignin deposition nor an increase in SPOX activity was found. Increased lignification is believed to be responsible for growth reduction in heavy metal-treated plants [63]. Our results indicate that in the Ni-treated cucumber plants, restriction of the leaf blade growth might be attributed, at least partly, to an increased lignification; however, other processes had to be involved in the root growth inhibition.

## 4. Materials and Methods

### 4.1. Plant Material and Growth Conditions

Seeds of cucumber (*Cucumis sativus* L.) cv. Cezar were germinated for 6 days. Then the seedlings were transferred into diluted (1:4) Hoagland nutrient solution containing 10 µM Ni supplied as chloride. An equimolar concentration of Cl^-^ (in the form of NaCl) was added to the control nutrient solution instead of NiCl_2_. The pH of the nutrient solution was adjusted to 5.8. The plants were grown in a controlled-climate room at 24 °C and 175 µmol m^−2^ s^−1^ photosynthetic photon flux density (PPFD), with a 16 h photoperiod. After 14 days, the 1st leaves and roots were harvested. Samples were immediately frozen in liquid nitrogen and stored at −70 °C pending analysis.

### 4.2. Growth Parameters, Ni Contents, and Relative Water Content

The fresh weight (FW) of the leaves and roots was measured immediately after harvesting. Dry weight (DW) was measured after oven-drying of the leaf/root sample for 48 h at 105 °C. Relative water content (RWC) was determined according to Smart and Bingham [64] and calculated as RWC [%] = [(FW − DW)/(FSW − DW)] × 100. Fully saturated weight (FSW) was determined after floating of the leaf/root sample in distilled water for 4 h at 20 °C in darkness.

Nickel contents in the cucumber tissues were determined by atomic absorption spectrometry. The dried tissue (100 mg) was digested in 5 cm^3^ 69% HNO_3_ using a microwave mineralizer (UniClever, Plazmatronika, Wroclaw, Poland). Nickel contents were determined using a Varian SpectrAA 300 spectrometer (Varian Australia Pty. Ltd., Mulgrave, Australia) equipped with a deuterium lamp for background correction and an air/acetylene flame at 232 nm. The standards of Ni (T.J.Baker, Phillipsburg, NJ, USA) were used for calibration. Nickel contents were expressed in µg per g DW.

### 4.3. Photosynthetic Pigment Concentrations and Chlorophyll a Fluorescence

For determination of photosynthetic pigment concentrations, the leaves were homogenized twice with 80% acetone and centrifuged (33,987× *g*, 15 min). After measurement of absorbance of the supernatants at 663 nm, 647 nm, and 470 nm, chlorophyll *a*, chlorophyll *b,* and total carotenoid contents were calculated according to the method of Wellburn [65].

Maximum photochemical efficiency of PSII (QY), fluorescence decline ratio (Rfd), and non-photochemical quenching (NPQ) were estimated using a portable fluorometer FluorPen FP110-LM/D (Photon Systems Instruments, Drásov, Czech Republic). Prior to measurements, the plants were dark-adapted for 20 min. Five measurements were performed per leaf blade, and the results were averaged.

### 4.4. HPLC Analysis

The plant tissue was homogenized in a mortar in an 80% methanol solution (1:5 *w*/*v*), then the homogenate was centrifuged (33,987× *g*, 15 min, 4 °C). The obtained supernatant was poured off and the pellet was extracted with 90% methanol solution and centrifuged again (33,987× *g*, 15 min, 4 °C). After pouring off the supernatant, the pellet was extracted with 100% methanol and centrifuged (33,987× *g*, 15 min, 4 °C). The mixture of 3 obtained supernatants was poured into round-bottom flasks and evaporated to dryness under vacuum at 65 °C. The residue was re-dissolved in hot distilled water and poured into centrifuge tubes. The procedure was repeated 3 times, and the supernatants were centrifuged (33,987× *g*, 15 min, 4 °C). The obtained extracts were supplemented with water to the same volume (6 cm^3^) and divided into two parts: for the determination of free and conjugated phenolic compounds. To determine the pool of free phenolic compounds, 0.5 cm^3^ of the extract was taken, poured into an Eppendorf tube, and mixed with 1 cm^3^ of HPLC-grade methanol. To determine the content of free SA, the extracts were adjusted to pH~2, and the resulting precipitate was centrifuged (33,987× *g*, 15 min) and extracted three times with a mixture of cyclopentane, ethyl acetate, and isopropanol (50:50:1), each time shaking and collecting the upper organic layer in round-bottom flasks. Then the extracts were evaporated in a vacuum evaporator at 55 °C, and the resulting precipitate was dissolved in 70% methanol with 0.5% formic acid. Until analysis, the samples were stored in a freezer at −18 °C.

To release phenolic compounds from the conjugated form, the extracts were subjected to acid hydrolysis. The extract (3 cm^3^) was hydrolyzed in 4 M HCl at 80 °C for 1.5 h. Then the samples were cooled and adjusted to pH ~ 2 with 30% NaOH, poured into centrifuge tubes, and centrifuged. To determine the content of phenolic compounds released from the conjugated form, 0.5 cm^3^ of the hydrolyzed extract was taken and poured into an Eppendorf tube, and 1 cm^3^ of methanol (HPLC-grade) was added. Extraction of the released salicylic acid was carried out with a mixture of cyclopentane, ethyl acetate, and isopropyl alcohol (50:50:1) in the same way as in the case of free SA. Until assayed, the samples were stored in a freezer at −18 °C.

Chromatographic analysis of the phenolic compounds was carried out using an HPLC system (Summit x2 Dual-Gradient System, Dionex, Sunnyvale, CA, USA) equipped with a photodiode-array detector (PDA100 DAD) and a fluorescence detector (RF-2000). Phenolic compounds present in the extracts were separated on an RP-C18 column (aQ Hypersil GOLD, 250 × 4.6 mm, 5 mm) joined with a guard column (GOLD aQ Drop-In guards, 10 × 4 mm^2^, 5 mm, Polygen, Gliwice, Poland) at 25 °C. The injection volume of the analyzed samples was 20 mm^3^. A mobile phase composed of water (A) and methanol (POCH, Gliwice, Poland) (B), both with 0.1% formic acid (Sigma-Aldrich, Saint Louis, MO, USA), was used. The linear gradient was started after 2 min of isocratic elution with 5% B, increasing slowly over 30 min to 55% B, followed by 5 min of isocratic elution. Between 35 and 45 min, the concentration of phase B increased to 70% followed by 5 min isocratic elution. Then between 50 and 52 min, the gradient was returned to the initial 5% B, and the column was recalibrated for the next 3 min. The flow rate was 1 cm^3^ min ^−1^. The absorbance was measured at 235, 280, 325, and 375 nm and the fluorescence at 420 nm (excitation 270 nm, emission 420 nm). Phenolic compounds in the extracts were identified by comparing the retention times and online UV absorption spectra of the analyzed samples with the respective data obtained from reference standards (Sigma-Aldrich, Saint Louis, MO, USA). Quantification was based on calibration curves for standards of phenolic compounds covering the range 5–200 mg cm^−3^; the linearity of the calibration curve was verified by the correlation coefficient (r^2^ ≥ 0.9994). Prior to total phenolic content analysis, obtained water extracts were centrifuged (33,987× *g*, 20 min); additionally, before HPLC analysis, supernatants were filtered through a 45 µm syringe filter (Millipore, Billerica, MA, USA). The content of phenolic compounds was expressed as μg per g of FW.

### 4.5. Enzyme Extraction and Assays

The plant tissue was homogenized (1:10 *w*/*v*) in an ice-cold mortar using 50 mM sodium phosphate buffer pH 7.0 containing 1 mM EDTA. After centrifugation (33,987× *g*, 15 min, 4 °C) the supernatant was used for the determination of GPOX, SPOX, FPOX, QPOX, PAL, PPOC, and PPOL activities.

GPOX activity was measured by the method of Maehly and Chance [66]. The assay mixture contained 50 mM sodium acetate buffer pH 5.6, 5.4 mM guaiacol, 15 mM $H_{2}O_{2}$, and enzyme extract. The increase in absorbance due to the oxidation of guaiacol to tetraguaiacol (ε = 26.6 mM^−1^ cm^−1^) was monitored at λ = 470 nm. GPOX activity was expressed in micromoles of tetraguaiacol formed per 1 min per 1 mg of protein (µmol min^−1^ mg^−1^ protein).

SPOX activity was measured by the method of Imberty et al. [67]. The reaction mixture consisted of 50 mM phosphate buffer (sodium salt) pH 6.0, 0.11 mM $H_{2}O_{2}$, enzyme extract, and 29 μM syringaldazine, the addition of which initiated the reaction. At the same time, reference samples without $H_{2}O_{2}$ were prepared. The increase in absorbance was determined at a wavelength of λ = 530 nm for 4 min. Enzyme activity was calculated using the molar absorption coefficient for the syringaldazine oxidation product, ε = 27 mM ^−1^ cm^−1^. SPOX activity was expressed in micromoles of syringaldazine oxidized per 1 min per 1 mg of protein (µmol min^−1^ mg^−1^ protein).

FPOX activity was determined based on the change in absorbance during oxidation of ferulic acid (ε = 11.3 mM^−1^ cm^−1^) according to a modified method described by Takahama [68]. The reaction mixture contained 50 mM phosphate buffer (sodium salt) pH 6.0, 0.1 mM $H_{2}O_{2}$, enzyme extract, and 0.14 mM ferulic acid. Absorbances were measured on a spectrophotometer at a wavelength of λ = 310 nm for 4 min. FPOX activity was expressed in micromoles of ferulic acid oxidized per 1 min per 1 mg of protein (µmol min^−1^ mg^−1^ protein).

QPOX activity was determined based on the change in absorbance during quercetin oxidation (ε = 17,5 mM^−1^ cm^−1^) [69] according to the modified method of Takahama and Hirota [70]. The reaction mixture contained 50 mM phosphate buffer (sodium salt) pH 7.0, 50 µM quercetin, enzyme extract, and 50 µM $H_{2}O_{2}$. Absorbances were measured on a spectrophotometer at λ= 380 nm for 4 min. QPOX activity was expressed in micromoles of quercetin oxidized per 1 min per 1 mg of protein (µmol min^−1^ mg^−1^ protein).

PAL activity was determined spectrophotometrically according to the modified method of Kolupaev et al. [71]. The reaction mixture contained borate buffer pH 8.8, enzyme extract, and 50 mM L-phenylalanine. Enzyme activity was measured at λ = 290 nm, and then the samples were incubated at 37 °C. After an hour, 5 M HCl was added and the absorbance was determined again. Enzyme activity was calculated using the molar absorption coefficient for the formed product (*trans*-cinnamic acid), ε = 9 mM^−1^ cm^−1^. PAL activity was expressed in nanomoles of *trans*-cinnamic acid formed per 1 min per 1 mg of protein (nmol min^−1^ mg^−1^ protein).

Activities of polyphenol oxidases: PPOC and PPOL were assayed according to the modified method by Dörnenburg and Knorr [72]. For the PPOC assay, the reaction mixture (2 cm^3^) consisted of 0.05 M sodium acetate buffer pH 5.6, 11.35 mM pyrocatechol, and the enzyme extract. After 2 h of incubation at 30 °C, the absorbance was measured at 420 nm. For the PPOL assay, the reaction mixture (2 cm^3^) consisted of 0.05 M sodium acetate buffer pH 5.6, 6.9 mM 1,4-phenylenediamine dihydrochloride, and the enzyme extract. After 2 h of incubation at 30 °C, the absorbance was measured at 530 nm. The activities of PPOC and PPOL were expressed as a change in absorbance per 1 min per 1 mg of protein.

### 4.6. Native Polyacrylamide Gel Electrophoresis (PAGE)

The electrophoretic separation of POX isoenzymes was carried out in a polyacrylamide gel under non-denaturing conditions. The plant tissue was homogenized (1:10 *w*/*v*) in 50 mM sodium phosphate buffer pH 7.0 containing 1 mM EDTA and 1% PVP. The samples were then centrifuged (33,987× *g*, 15 min, 4 °C). The supernatant was mixed with loading buffer (40% sucrose, 0.01% bromophenol blue) in a ratio of 2:1 *v*/*v*.

The electrophoretic separation of POX isoenzymes was carried out in an 8% polyacrylamide gel. The polyacrylamide gel was prepared in two stages. In the first stage, a separating gel was prepared, which contained deionized water, 30% acrylamide/bisacrylamide, 1.5 M Tris-HCl buffer pH 8.8, 10% ammonium persulfate, and 2.8 mM N,N,N′,N′-tetramethylethylenediamine (TEMED). To remove any air bubbles, an aqueous solution of isobutanol was applied to the separating gel. In the second stage, a thickening gel was prepared at a concentration of 4%, to which 0.5 M Tris-HCl buffer pH 6.8 was added instead of 1.5 M Tris-HCl buffer. The polymerized gels were placed in an electrophoresis apparatus (Mini-PROTEAN Tetra Cell, BioRad, Hercules, CA, USA) in 5x concentrated electrophoresis buffer (125 mM Tris and 1.25 M glycine, pH 8.3). Electrophoresis was carried out at a current intensity of 20 mA under a voltage of 100/165 V at a temperature of 4 °C until the migrating dye front reached the lower limit of the gel.

For the identification of POX isoforms, gels were stained for 30 min in 100 mM acetate buffer pH 5.0 with 0.2 M guaiacol at room temperature. $H_{2}O_{2}$ was then added to a final concentration of 0.1% and incubated until bands appeared. Stained gels were analyzed using a Gel Doc EZ System gel documenter (BioRad, Hercules, CA, USA).

### 4.7. Lignin Detection

For the detection of lignin, a mixture of phloroglucinol–HCl (3% phloroglucinol in ethanol/concentrated HCl, 2:1, *v*/*v*) was used [73]. In the case of leaves, chlorophyll was removed using a mixture of methanol–acetic acid (9:1, *v*/*v*) before lignin detection [74]. Finally, the leaf and root scans were taken.

### 4.8. Statistical Analysis

The results are means of 3 independent experiments. For fresh weight estimation, 4 plants were analyzed per treatment in each experiment, so the data present the means of 12 observations. For the other parameters, 1–3 samples were analyzed per treatment in each experiment (n = 3–9). The significance of differences between Ni-treated seedlings and the control was estimated using one-way ANOVA followed by the Fisher’s least significant difference (LSD) test (*p* < 0.05). Sample variability was given as the standard deviation of the mean. The statistical analysis was carried out using Statistica 13.1 software.

## 5. Conclusions

Our study revealed the Ni-induced remodeling of the phenolics profile in the cucumber leaf and root. The changes in the content of individual compounds are suggested to result rather from conversions of the compounds constitutively present in the tissues than from de novo synthesis. The Ni-triggered release of free phenolics from their conjugated forms may lead to improvement of their antioxidant and chelating properties.

## Figures and Tables

**Table 1:** Effect of Ni treatment on growth, Ni accumulation, and the relative water content in the cucumber leaf and root. Data represent mean values ± SD (n = 6–12); * indicates values that differ significantly from the control at *p* < 0.05.

Treatment	Fresh weight [mg]	Ni content [μg g^−1^ DW]	RWC [%]
**Control leaf**	284.3 ± 58.9	1.98 ± 0.75	89.84 ± 0.22
**Ni leaf**	133.6 ± 43.3 *	292.85 ± 65.81 *	87.62 ± 1.33
**Control root**	533.47 ± 104.87	4.38 ± 0.42	95.16 ± 0.40
**Ni root**	221.24 ± 58.77 *	592.89 ± 82.12 *	94.97 ± 0.48

**Table 2:** Effect of Ni treatment on free and conjugated phenol contents in the cucumber leaf. Data represent mean values ± SD (n = 3); * indicates values that differ significantly from the control at *p* ˂ 0.05; numbers highlighted in 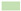/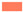 coloration indicate increases/decreases compared to the control.

Metabolite [µg g^−1^ FW]		Free		Conjugated		Free/Total [%]	
		Control	Ni	Control	Ni	Control	Ni
**Phenolic acids**	*trans*-3-hydroxycinnamic acid	0	33.75 ± 8.72	0	4.52 ± 1.25	-	91.0 ± 8.65
	ellagic acid	37.06 ± 2.97	36.34 ± 4.8	64.49 ± 5.57	30.75 ± 2.27 *	36.5 ± 1.44	54.2 ± 3.47 *
	4-hydroxybenzoic acid	13.91 ± 1.41	12.52 ± 1.07	73.63 ± 6.72	25.15 ± 2.76 *	15.9 ± 0.55	33.2 ± 0.97 *
	ferulic acid	4.81 ± 0.41	4.82 ± 0.67	6.43 ± 0.47	5.81 ± 1.06	42.8 ± 0.62	45.3 ± 4.82
	chlorogenic acid	16.64 ± 2.2	13.88 ± 1.74	3.43 ± 0.89	2.96 ± 0.90	82.9 ± 2.69	82.4 ± 3.55
	*trans*-cinnamic acid	4.64 ± 0.49	4.67 ± 0.47	1.04 ± 0.53	0.69 ± 0.28	81.7 ± 9.36	89.8 ± 4.23 *
	*p*-coumaric acid	18.62 ± 2.42	24.6 ± 2.65 *	9.32 ± 1.67	16.67 ± 3.42 *	66.6 ± 1.86	59.6 ± 7.91
	1,3-dicaffeoylquinic acid	6.68 ± 1.15	5.42 ± 0.65	1.25 ± 0.32	0.97 ± 0.30	87.2 ± 2.99	84.8 ± 3.55
	gallic acid	0	0.27 ± 0.05	0	1.43 ± 0.31	-	15.9 ± 1.48
	salicylic acid	0.15 ± 0.02	0.29 ± 0.09	0.44 ± 0.05	0.57 ± 0.08	25.5 ± 3.16	33.3 ± 7.52
	**Total**	**102.51**	**136.56 (133%)**	**160.02**	**89.5 (56%)**	**39.0**	**60.8 (156%)**
**Flavonoids**	naringenin	0.89 ± 0.22	0.17 ± 0.04 *	0.12 ± 0.06	0.03 ± 0.008 *	88.1 ± 5.47	85.0 ± 7.14
	3-hydroxyflavone	56.26 ± 4.58	56.28 ± 4.35	60.79 ± 4.35	62.59 ± 8.32 *	48.1 ± 0.70	47.3 ± 2.24
	(+)-catechin	59.93 ± 5.76	33.41 ± 2.65 *	102.59 ± 4.85	70.45 ± 2.89 *	36.9 ± 1.21	32.2 ± 1.05
	(−)-epicatechin	21.39 ± 3.08	36.92 ± 4.66 *	3.48 ± 1.44	78.42 ± 5.99 *	96.5 ± 4.82	32.0 ± 1.45 *
	cyanidin	22.54 ± 4.66	9.75 ± 1.37 *	0.65 ± 0.16	1.88 ± 0.56 *	83.8 ± 5.02	86.7 ± 6.93
	quercetin	0	0	15.19 ± 1.99	13.44 ± 1.91	-	-
	luteolin	0	0	33.37 ± 1.63	31.65 ± 3.34	-	-
	hesperetin	2.39 ± 0.48	1.93 ± 0.36	15.23 ± 1.41	19.56 ± 1.17	13.6 ± 2.11	9.0 ± 1.21 *
	flavone	6.99 ± 0.94	19.17 ± 1.71 *	9.00 ± 1.02	23.02 ± 1.65 *	43.7 ± 3.33	45.4 ± 1.26
	procyanidin B2	19.35 ± 3.65	43.05 ± 2.35 *	36.48 ± 3.00	0.77 ± 0.93 *	34.7 ± 4.31	93.3 ± 3.59 *
	**Total**	**189.74**	**200.68 (106%)**	**276.91**	**301.81 (109%)**	**40.6**	**39.8 (98%)**

**Table 3:** Effect of Ni treatment on free and conjugated phenol contents in the cucumber root. Data represent mean values ± SD (n = 3); * indicate values that differ significantly from the control at *p* ˂ 0.05; numbers highlighted in 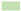/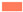 coloration indicate increases/decreases compared to the control.

Metabolite [µg g^−1^ FW]		Free		Conjugated		Free/Total [%]	
		Control	Ni	Control	Ni	Control	Ni
**Phenolic acids**	gallic acid	59.9 ± 3.84	6.36 ± 1.17 *	1.05 ± 0.55	3.47 ± 0.31 *	99.1 ± 7.32	64.7 ± 5.76 *
	protocatechuic acid	21.92 ± 2.99	54.81 ± 4.99 *	4.32 ± 1.59	1.60 ± 0.48 *	83.5 ± 6.55	97.0 ± 0.69 *
	α-resorcylic acid	4.56 ± 0.47	8.07± 0.84 *	0.45 ± 0.08	0.61 ± 0.51	91.0 ± 1.62	93.0 ± 0.51
	*trans*-3-hydroxycinnamic acid	51.02 ± 2.6	22.73 ± 0.33 *	23.40 ± 2.42	28.19 ± 1.54 *	68.6 ± 1.78	44.6 ± 1.68 *
	sinapic acid	147.37 ± 2.98	189.9 ± 4.36 *	80.49 ± 5.23	48.03 ± 4.87 *	64.7 ± 1.52	79.8 ± 1.31 *
	ellagic acid	15.67 ± 0.86	21.18 ± 2.11 *	124.15 ± 5.36	60.88 ± 2.07 *	11.2 ± 0.99	25.8 ± 1.78 *
	rosmarinic acid	24.91 ± 1.93	24.84 ± 1.13	25.89 ± 3.18	18.52 ± 3.85	49.0 ± 4.02	57.3 ± 5.77
	4-hydroxybenzoic acid	5.46 ± 1.11	6.7 ± 1.06	21.45 ± 0.8	42.21 ± 2.73 *	20.3 ± 2.88	13.7 ± 1.12 *
	ferulic acid	1.15 ± 0.17	11.52 ± 0.54 *	31.51 ± 0.91	27.47 ± 0.56 *	3.5 ± 0.41	29.5 ± 0.64 *
	β-resorcylic acid	0.1 ± 0.01	0.07 ± 0.004 *	0.81 ± 0.01	0.43 ± 0.04 *	11.0 ± 1.09	13.7 ± 1.68
	chlorogenic acid	0.18 ± 0.03	0.75 ± 0.04 *	0.01 ± 0.001	0.08 ± 0.05 *	94.7 ± 2.54	91.5 ± 2.28
	vanillic acid	0.44 ± 0.01	0.43 ± 0.12	0.09 ± 0.08	0.07 ± 0.05	83.0 ± 13.13	86.0 ± 12.09
	trans-cinnamic acid	0.32 ± 0.02	0.88 ± 0.06 *	1.51 ± 0.03	1.00 ± 0.06	17.5 ± 0.92	46.8 ± 1.46 *
	*p*-coumaric acid	0.27 ± 0.05	2.14 ± 0.17 *	0.48 ± 0.05	0.62 ± 0.12	36.0 ± 5.70	77.5 ± 4.73 *
	*o*-coumaric acid	0.5 ± 0.02	1.29 ± 0.11 *	0.06 ± 0.01	1.65 ± 0.28 *	89.3 ± 1.88	43.9 ± 3.75 *
	salicylic acid	0.22 ± 0.08	0.16 ± 0.05	0.50 ± 0.12	0.25 ± 0.02	30.8 ± 9.56	39.0 ± 6.84
	**Total**	**333.99**	**351.83 (105%)**	**316.17**	**235.08 (74%)**	**51.4**	**59.9 (117%)**
**Flavonoids**	naringenin	2.86 ± 0.08	1.43 ± 0.11 *	2.12 ± 0.18	1.71 ± 0.16 *	57.4 ± 2.66	45.5 ± 0.44 *
	3-hydroxyflavone	25.26 ± 2.25	22.95 ± 2.22	32.90 ± 2.59	30.58 ± 1.82	43.4 ± 1.93	42.9 ± 2.10
	(+)-catechin	40.21 ± 3.07	45.65 ± 4.18	98.03 ± 5.81	48.36 ± 6.14 *	29.1 ± 2.72	48.6 ± 4.84 *
	(−)-epicatechin	24.74 ± 1.59	20.06 ± 2.71	20.70 ± 1.17	55.26 ± 5.84 *	54.4 ± 2.0	26.6 ± 3.73 *
	(−)-epigallocatechin	53.08 ± 3.57	15.75 ± 1.6 *	0.70 ± 0.28	49.45 ± 3.5 *	98.7 ± 0.53	24.2 ± 2.65 *
	cyanidin	5.75 ± 0.38	12.36 ± 1.89 *	25.52 ± 0.61	21.06 ± 3.03	18.4 ± 1.28	37.1 ± 3.33 *
	quercetin	0	0	1.94 ± 0.1	5.24 ± 0.15 *	-	-
	myricetin	21.48 ± 2.39	17.03 ± 2.32	4.32 ± 0.39	2.49 ± 0.33 *	83.2 ± 9.58	87.3 ± 7.91
	luteolin	1.59 ± 0.31	0	9.22 ± 0.86	6.38 ± 0.93	14.7 ± 2.49	-
	**Total**	**174.97**	**135.23 (77%)**	**195.45**	**220.53 (113%)**	**47.2**	**38.0 (81%)**
**Other**	*p*-benzoquinone	4.8 ± 0.24	4.91 ± 0.68	22.99 ± 3.9	9.04 ± 0.89 *	17.3 ± 1.94	35.2 ± 3.88 *
	pyrocatechol	4.39 ± 0.55	9.25 ± 0.72 *	20.96 ± 2.5	10.28 ± 2.33 *	17.3 ± 2.38	47.3 ± 6.65 *
	**Total**	**9.19**	**14.16 (154%)**	**43.95**	**19.32 (44%)**	**17.3**	**42.3 (244%)**

## Data Availability

The original data presented in the study are openly available in [ijms-26-01237 data_Gajewska et al.] at [https://uniwersytetlodzki-my.sharepoint.com/:x:/g/personal/ewa_gajewska_biol_uni_lodz_pl/EZrT-h3PRbxCkb7baoQocbYBaqzNNh9yuq247qIzxoNbtA, accessed on 10 January 2025].

## Abbreviations

DW	Dry weight
FW	Fresh weight
FSW	Fully saturated weight
FPOX	Peroxidase assayed with ferulic acid
GPOX	Peroxidase assayed with guaiacol
$H_{2}O_{2}$	Hydrogen peroxide
NPQ	Non-photochemical quenching
PAL	Phenylalanine ammonia lyase
POX	Peroxidase
PPFD	Photosynthetic photon flux density
PPOC	Catecholase
PPOL	Laccase
PS II	Photosystem II
Rfd	Fluorescence decline ratio
ROS	Reactive oxygen species
RWC	Relative water content
SA	Salicylic acid
SPOX	Peroxidase assayed with syringaldazine
QPOX	Peroxidase assayed with quercetin
QY	Maximum photochemical efficiency of PS II
